# Identification of blood meal sources in the main African malaria mosquito vector by MALDI-TOF MS

**DOI:** 10.1186/s12936-016-1152-6

**Published:** 2016-02-13

**Authors:** Sirama Niare, Jean-Michel Berenger, Constentin Dieme, Ogobara Doumbo, Didier Raoult, Philippe Parola, Lionel Almeras

**Affiliations:** Unité de Recherche en Maladies Infectieuses et Tropicales Emergentes (URMITE), UM63, CNRS 7278, IRD 198 (Dakar, Sénégal), Inserm 1095, Faculté de Médecine, Aix Marseille Université, 27 bd Jean Moulin, 13385 Marseille cedex 5, France; Malaria Research and Training Center, DEAP/FMOS, UMI 3189, University of Science, Techniques and Technology, Bamako, Mali

**Keywords:** MALDI-TOF mass spectrometry, Blood meal source, *Anopheles*, Epidemiology, Outbreak, Surveillance

## Abstract

**Background:**

The identification of blood meal sources in malaria vectors is critical to better understanding host/vector interactions and malaria epidemiology and control. Currently, the identification of mosquito blood meal origins is based on time-consuming and costly techniques such as precipitin tests, ELISA and molecular tools. Although these tools have been validated to identify the blood meal and trophic preferences of female *Anopheles* mosquitoes, they present several limitations. Recently, matrix-assisted, laser desorption/ionization time-of-flight mass spectrometry (MALDI-TOF MS) was successfully used as a quick and accurate tool for arthropod identification, including mosquitoes. The aim of the present work was to test whether MALDI-TOF MS could also be applied to identification of blood meal sources from engorged mosquitoes.

**Methods:**

Abdomen proteins extracted from *Anopheles gambiae* (*stricto* sensu, S molecular form) that were either unengorged or artificially engorged on seven distinct types of vertebrate blood (human, horse, sheep, rabbit, mouse, rat, dog) were submitted for MALDI-TOF MS.

**Results:**

The comparison of mass spectrometry (MS) spectra from mosquito abdomens collected 1 h post-feeding, were able to discriminate blood meal origins. Moreover, using *Aedes albopictus* specimens, abdominal protein MS spectra from engorged mosquitoes were found specific to host blood source and independent of the mosquito species. A sequential analysis revealed stability of mosquito abdominal protein spectra up to 24 h post-feeding.

**Conclusions:**

These results indicate that MALDI-TOF MS could determine feeding patterns of freshly engorged mosquitoes up to 24 h post-blood meal. The MALDI-TOF MS technique appears to be an efficient tool for large epidemiological surveillance of vector-borne diseases and outbreak source identification.

## Background

Malaria is a parasitic infection caused by a protozoan of the *Plasmodium* genus, with six species infecting humans, causing diseases and creating a burden on public health systems: *Plasmodium falciparum, P. vivax, P. malariae, P. ovale wallikeri, P. ovale curtisi,* and *P. knowlesi*. The malaria parasite is responsible for about 198 million cases worldwide and about 584,000 deaths per year [1]. Falciparum malaria remains the main cause of morbidity and mortality in Africa [1]. The malaria parasite is transmitted during blood meals of infected female mosquitoes of the *Anopheles* genus. In the world, 470 *Anopheles* species have been described, among which around 50 species are capable of malaria transmission [2]. To control this vector-borne disease, a better understanding of malaria transmission dynamics is indispensable. To this end, precise identification of anopheline fauna in a specific area is essential for providing information about vector species involved in malaria transmission, planning effective control measures and monitoring their impact [3]. In addition, determining the degree of contact between *Anopheles* malaria vectors and host populations, notably by analysing the range of hosts of a given *Anopheles* species, is crucial to assessing risk of human exposure and changes in *Anopheles* trophic preference [4]. The combination of the *Anopheles* fauna dynamics and their respective trophic behaviours are important factors affecting the risk of malaria transmission and human mosquito bite exposure.

Today, mosquito identification is mainly based on the detection of morphological characters using taxonomic keys or on sequencing of targeted genes [5, 6]. However, both of these approaches present several limitations, such as the requirement of entomological skills or the lack of DNA barcoding for *Anopheles* population identification. To circumvent these limitations, the matrix-assisted, laser desorption/ionization time-of-flight mass spectrometry (MALDI-TOF MS) was recently evaluated for arthropod identification [7, 8]. The application of MALDI-TOF MS to mosquito populations demonstrated that this innovative method could unambiguously distinguish not only mosquito species, but also specimens from the same complex and molecular forms of *Anopheles gambiae stricto* sensu (*s.s.)*(S molecular form) [9–11]. Recently, this proteomic tool was applied to *Anopheles* identification at immature stages [12]. The low-cost, rapidity and robustness of MALDI-TOF MS made this tool a reliable method for arthropod identification.

To obtain an overview of human risk exposure to *Anopheles* bites, the identification of blood meal sources in recently blood-fed mosquito vectors is an alternative for assessment of hosts/*Anopheles* contacts. The identification of vertebrate feeding sources and host preferences is essential for studying the transmission dynamics of vector-borne pathogens. Host-feeding patterns and preferences vary according to environmental factors, including host availability and abundance, flight behaviour and feeding periodicity of mosquitoes [13]. The estimation of the human blood index (HBI), corresponding to the proportion of blood meals derived from humans by mosquito vectors, is an important component of vector capacity, as a proxy measurement of malaria transmission [14]. Traditional serological techniques, including precipitin tests and enzyme-linked immunosorbent assays (ELISA), have been used to identify hosts from diverse insect vectors [15, 16]. Although the use of these methods has provided valuable information, it has several limitations, including the difficulty of obtaining specific antisera against a broad diversity of host species and thereby failing to detect any host being investigated. To solve these limitations, researchers have progressively incorporated molecular biology approaches to identify blood sources at the species level [17]. Although molecular tools have been proven capable of identifying trophic preferences of female mosquitoes from the *Anopheles* genus, they present several limitations, notably their labour-intensive and expensive nature. Successful identification of hosts by PCR-based methods are directly linked to the quality and quantity of the vertebrate’s DNA contained in the mosquito abdomen [18, 19]. After feeding, the digestion of the blood meal occurs in the insect gut, leading to a quick degradation of host DNA. Therefore, 30 to 36 h following mosquito blood meals, the success rate of identification of blood meal sources with molecular biology falls to less than 50 % [20]. Similar results were previously reported by Oshaghi and collaborators using another target gene sequences [21]. Moreover, gene sequencing remains time consuming, technically demanding and expensive. Indeed, to overcome the limitations of immunological and molecular methods, the development of an innovative, robust method that is cost effective, rapid and reliable in identifying vertebrate sources of mosquito blood meals was needed.

Based on the success of MALDI-TOF MS for arthropod identification [8, 9], the goal of the present work was to test the usefulness of this proteomic approach for the rapid identification of blood meal sources from engorged mosquitoes. For this purpose, abdominal proteins extracted from *An. gambiae s.s.* that were both unengorged and artificially engorged on seven distinct types of vertebrate blood (human, horse, sheep, rabbit, mouse, rat, dog) were submitted for MALDI-TOF MS. MS spectra from mosquito abdomens were compared to research-specific protein profiles according to blood meal host origins, and kinetic analysis assessed protein spectra stability according to post-feeding delay. The advantages, limitations and alternative strategies to improve the definition of mosquito feeding patterns by MALDI-TOF MS were discussed.

## Methods

### Ethic statements and good laboratory practices

All the procedures adopted in this study were approved by the human and animal ethics committees of the Institutional Animal Care and Committee of Aix-Marseille University. The animal blood (mice, rabbit, horse, sheep, rat, dog) was provided by local animal houses and was handled according to the rules of Décret no. 8 87–848 (October 19, 1987, Paris). Human blood was obtained from Etablissement Français du Sang (EFS), using existing conventions between the laboratory and EFS. EFS blood samples were processed and stored using Good Laboratory Practices from the World Health Organization (WHO) and documents on blood samples handling procedures [22]. Mosquitoes were raised using the International Conference on Harmonization/Good Laboratory Practices (ICH/GLP) procedures. Laboratory technicians and students were trained and certified in animal- and insectaria-based experiments.

### Mosquitoes

*Anopheles gambiae s.s.* (S molecular form) and *Aedes albopictus* mosquitoes were raised at the laboratory using standard methods with temperature of 26 ± 1 °C, a relative humidity of 80 ± 10 % and a 12-hour photoperiod in stand-alone incubators (Panasonic cooled incubator) [23]. Larvae were raised until the pupal stage in trays containing 1 L of distilled water supplemented with fish food (TetraMinBaby, Tetra Gmbh, Herrenteich, Germany). Pupae were collected daily and transferred into a mosquito cage (Bug Dorm 1, Bioquip products). Adults were fed with a 10 % glucose solution until the day of the experiment. Three days after emergence, female adult mosquitoes were artificially fed through a Parafilm-membrane (Hemotek membrane feeding systems, Discovery Workshops, UK) using fresh heparinized vertebrate blood (i.e., human, horse, sheep, mouse, rat, rabbit or dog) for 2 h. Engorged mosquitoes were transferred into another cage and were maintained in standard conditions with 10 % sucrose solution on cotton. Five engorged females were harvested at one, 12, 24, 48 and 60 h post-blood feeding for kinetic analysis of the blood meal sources. Specimens were anaesthetized at −20 °C for 10 min prior dissections. At the same time, abdomens from specimens receiving only a 10 % sucrose solution were collected and used as controls. Each mosquito abdomen was separated from the thorax using a sterile scalpel for each sample. The dissected abdomens, placed in individually labelled vials, were either immediately processed or stored at −20 °C.

### Preservation methods

Seventy-five *An. gambiaes.s.* mosquitoes engorged on human blood were harvested 12 h post-blood feeding and were preserved either at −20 °C (frozen group), in ethanol at 70 % (v/v) at room temperature (RT) (alcohol group, n = 25) or placed in tubes containing silica gel and cotton at RT (dry group, n = 25). For each preservation mode, the abdomens of five mosquitoes were separated from the thorax and tested individually in MALDI-TOF MS. Samples were submitted for MALDI-TOF MS at days 7, 14, 21, 30, and 60 according to their storing mode.

### Sample preparation

The abdomen of each mosquito was crushed in an Eppendorf tube containing 50 µL of water of high performance liquid chromatography (HPLC) quality. After a quick spin centrifugation to eliminate debris, 10 µL of supernatant was used for DNA extraction and 10µL for MALDI-TOF analysis.

### Molecular analysis

DNA extractions from individual mosquito abdomen samples were performed with the EZ1 DNA Tissue kit (Qiagen, Hilden, Germany) according to manufacturer recommendations. DNA extracted from unfed mosquitoes was used as a negative blood meal control. To assess the kinetic degradation of vertebrate blood DNA, a set of the primers specifically amplifying the vertebrate cytochrome c oxidase I gene (vCOI) was selected (vCOI_long forward: 5′-AAGAATCAGAATARGTGTTG-3′; vCOI_long reverse: 5′-AACCACAAAGACATTGGCAC-3′) [24]. To confirm that the absence of vCOI PCR product could be attributed to degradation of vertebrate blood DNA, a set of primer specifically amplifying a fragment of 710 bp of the mosquito Cytochrome c oxidase I gene (mCOI) was used as described previously [LCO1490 (forward):5′-GGTCAACAAATCATAAGATATTGG-3′; HC02198 (reverse): 5′-TAAACTTCAGGGTGACCAAAAAATCA-3′] [25]. Non-engorged mosquitoes were used as negative and positive controls for vCOI and mCOI PCR reactions, respectively. The PCR reaction contained 13 μl of sterile distilled water, 2.5 μl of tampon 5X Phusion HF Buffer, 2.5 μl of dNTPs, 0.5 μl of each primer, 0.25 μl of Taq, 1 μl of MgCl_2_ and 5 μl of extracted DNA. Reactions were amplified through 35 cycles with the following parameters: 10 min at 95 °C, 1 min at 95 °C, 1 min at 40 °C for vCOI, and 1 min at 52 °C for mCOI, 1.5 min at 72 °C, followed by a final extension step at 72 °C for 7 min. Amplifications were assessed by gel electrophoresis, using 2 % agarose/0.5 % TBE, stained with ethidium bromide. Some vCOI and mCOI positive PCR products were purified using the NucleoFast 96 PCR plate (Machery-Nagel EURL, France), as recommended by the manufacturer and sequenced using the same respective primers with the BigDye version 1-1 Cycle Ready Reaction Sequencing mix (Applied Biosystems, Foster City, CA, USA) and an ABI 3100 automated sequencer (Applied Biosystems) to control the amplified products. The sequences were assembled and analysed using the ChromasPro software (version 1.34) (Technelysium Pty. Ltd., Tewantin, Australia) and BLAST website [26].

### Sample loading on MALDI-TOF target plate

Ten µL of crushed abdomens were mixed with 20 µL of 70 % formic acid (v/v) and 20 µL of 50 % acetonitrile (v/v) (Fluka, Buchs, Switzerland) and centrifuged at 10,000 rpm for 20 s. One µL of supernatant of each sample was deposited on the MALDI-TOF target plate in quadruplicate (Bruker Daltonics, Wissembourg, France) and recovered with 1 µL of CHCA matrix solution composed of saturated α-cyano-4-hydroxycynnamic acid (Sigma, Lyon, France), 50 % acetonitrile (v/v), 2.5 % trifluoroacetic acid (v/v) (Aldrich, Dorset, UK) and HPLC-grade water. After drying for several minutes at RT, the target was introduced into Microflex LT MALDI-TOF Mass Spectrometer (Bruker Daltonics, Germany) for analysis. To control loading on mass spectra steel, matrix quality and MALDI-TOF apparatus performance, matrix solution was loaded in duplicate onto each MALDI-TOF plate with or without a bacterial test standard (Bruker protein Calibration Standard I).

### MALDI-TOF MS parameters

Protein mass profiles were acquired using a Microflex LT MALDI-TOF Mass Spectrometer (Bruker Daltonics, Germany), with detection in the linear positive-ion mode at a laser frequency of 50 Hz within a mass range of 2–20 kDa. The acceleration voltage was 20 kV, and the extraction delay time was 200 ns. Each spectrum corresponded to ions obtained from 240 laser shots performed in six regions of the same spot and automatically acquired using the AutoXecute of the Flex Control v.2.4 software (Bruker Daltonics). The spectrum profiles obtained were visualized with Flex analysis v.3.3 software and exported to ClinProTools version v.2.2 and MALDI-Biotyper v.3.0. (Bruker Daltonics, Germany) for data processing (smoothing, baseline subtraction, peak picking) and evaluation with cluster analysis.

### Spectra analysis and reference database creation

The reproducibility of MALDI-TOF MS spectra from mosquito specimens engorged on the same vertebrate and collected at different points in time was evaluated by comparing the average spectra obtained from the four spectra of each sample tested using the Flex analysis and ClinProTools 2.2 software (Bruker Daltonics). Specificity of MALDI-TOF MS spectra according to blood host origin was also analysed using the Flex analysis and ClinProTools 2.2 software (Bruker Daltonics). To create an MS database, reference spectra (MSP, Main Spectrum Profile) were created by combining the results of the spectra from at least three specimens per species that were either unfed or fed on the same vertebrate blood, and harvested at the same point in time by the automated function of the MALDI-Biotyper software v3.0. (Bruker Daltonics). MSP were created on the basis of an unbiased algorithm using information on the peak position, intensity and frequency.

### Blind tests

To determine the origin of blood meals, 158 MALDI-TOF MS spectra from abdominal protein extracts of laboratory-raised mosquitoes engorged on human, horse, sheep, mouse, rat, rabbit or dog blood collected at one, 12, 24, 36, 48, and 60 h post-blood feeding were evaluated against the reference database using the MALDI-Biotyper software v3.0. tool (Bruker Daltonics). The level of significance was determined using the log score values (LSVs) given by the MALDI-Biotyper software v.3.3. corresponding to a matched degree of signal intensities of mass spectra of the query and the reference spectra. LSVs ranged from zero to three. An LSV with the respective vertebrate name of the blood host origin was obtained for each spectrum of the samples tested blindly.

### Cluster analysis

Cluster analysis (MSP dendrogram) was performed based on comparison of the main spectra given by MALDI-Biotyper software and clustered according to protein mass profile (i.e., their mass signals and intensities). Several clustering analyses were performed to visualize the homogeneity level of MS spectra from specimens engorged on the same vertebrate host and collected at different points in time, but also to reveal MS spectra heterogeneity between specimens engorged on distinct vertebrate hosts and collected at the same point in time. The resulting MSP dendrogram portrays how samples are related to one another.

### Statistical analyses

Data were analysed by using GraphPad Prism software version 5.01 (GraphPad, San Diego, CA, USA). After verifying that the values in each group did not assume a Gaussian distribution, the Kruskal–Wallis test was used for multiple group comparisons. Two independent groups were compared by the Mann–Whitney U test. All differences were considered significant at *p* < 0.05.

## Results

### Kinetic detection of vertebrate DNA in mosquito abdomen post-blood feeding by molecular biology

A specific PCR assay based on the vertebrate vCO1 gene was performed on DNA extracted from unfed or human blood-fed *An. gambiae s.s.* and *Ae. albopictus* females collected at one, 12, 24, 36, 48, and 60 h post-feeding. Three specimens per point in time were tested. As expected, no vCO1 PCR product was detected for unfed *An. gambiae s.s.* and *Ae. albopictus*. DNA from human blood was detected in all fed mosquitoes of both species from 1 to 36 h post-feeding. At 48 h, only one of the three specimens tested yielded human DNA with lower band intensity for both mosquito species. Conversely, PCR products of vCO1 were negative for all samples collected 60 h post-feeding.

To ensure that the absence of vCO1 PCR products could not be attributed to failed DNA extraction, a specific PCR targeting of CO1 mosquitoes (mCO1) was performed on these same samples. A PCR product was detected in all samples tested regardless of their feeding status or the collection time, indicating that the absence of vCO1 products could not be attributed to DNA extraction failure. Taken together, these data confirmed that vertebrate DNA is degraded during the mosquito digestion process.

### Reproducibility, specificity and stability period of MALDI-TOF MS spectra from engorged mosquitoes

**Fig. 1:**
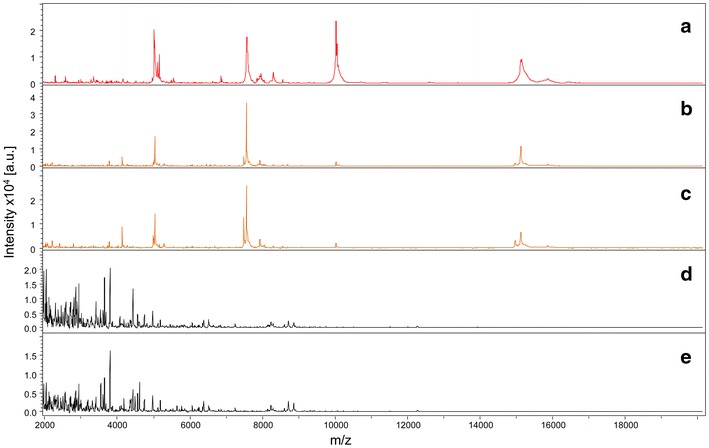
Assessment of MALDI-TOF MS spectra reproducibility from unfed and human blood fed *Anopheles gambiae S* abdomen protein extracts ranging from 2 to 20 kDa. Representative spectra from biological replicates of human blood (*a*), abdominal protein extracts of *An. gambiae S* engorged on human blood collected 1 h post-blood feeding (*b*,* c*) and abdominal protein extracts of unfed *An. gambiae S* (*d*,* e*). au arbitrary units; m/z mass-to-charge ratio

To test MS spectra reproducibility, five abdomens of *An. gambiae s.s.* that were unfed and fed on human blood and collected 1 h post-feeding were submitted for MALDI-TOF MS analysis. Comparison of the MS spectra with Flex analysis software indicated a high reproducibility of the protein profiles between biological replicates of abdominal protein extracts from blood-fed mosquitoes (Fig. 1). MS protein profiles were clearly visually distinct between fed and unfed specimens. Interestingly, the comparison of the MS protein profiles between human blood and *An. gambiae s.s.* specimens engorged on human blood indicated that several mass peaks were shared.

**Fig. 2:**
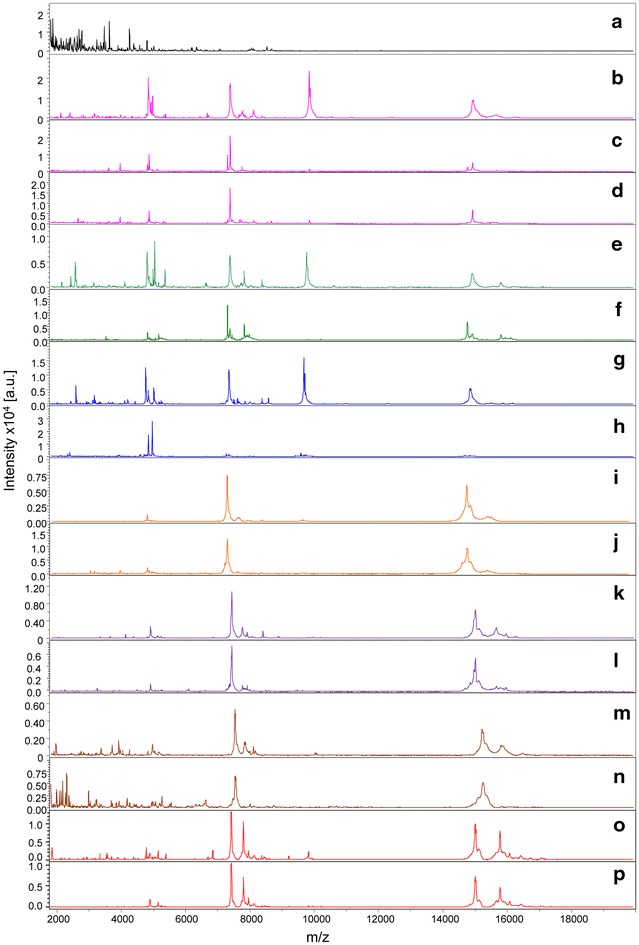
Comparison of MALDI-TOF MS profiles from mosquito specimens unfed or blood fed on seven distinct vertebrates. Representative spectra from human (*b*), horse (*e*), sheep (*g*), mouse (*i*), rat (*k*), rabbit (*m*) or dog (*o*) blood, and from abdominal protein extracts of unfed (*a*), fed *An. gambiae S* on human (*c*), horse (*f*), sheep (*h*), mouse (*j*), rat (*l*), rabbit (*n*) or dog bloods and *Ae. albopictus* fed on human blood (*d*). All mosquitoes were collected 1 h post-feeding. au arbitrary units; m/z mass-to-charge ratio

As MS protein profiles changed between fed and unfed mosquitoes, it was assessed whether the modification was specific to the blood host origin. To this end, a comparison of MS protein profiles from *An. gambiae s.s.* specimens engorged either on human, horse, sheep, mouse, rat, rabbit or dog blood and collected 1 h post-feeding was performed. As a control, the respective blood samples used for mosquitoes feeding from the five vertebrates were also submitted for MALDI-TOF MS analysis. Five samples per condition were tested. Visual comparison of MS profiles revealed that the spectra were not superimposable according to the origin of the blood meals (Fig. 2). Additionally, submission for MALDI-TOF MS analysis of abdominal protein extracts from *Ae. albopictus* specimens engorged on human blood and collected one hour post-feeding exhibited a profile similar to *An. gambiae s.s.* specimens engorged on human blood and collected at the same point in time (Fig. 2c, d).

**Fig. 3:**
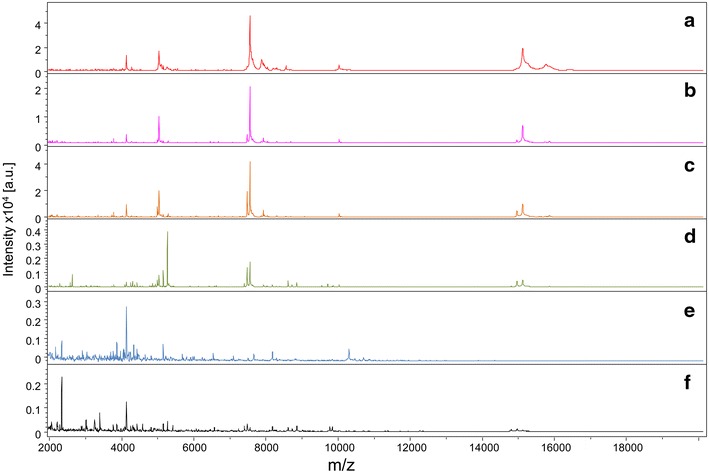
Alteration of MALDI-TOF MS profiles from *Anopheles gambiae S* specimens fed on human blood during digestion process. MS profiles from engorged *An. gambiae S* abdomens collected at 1 (*a*), 12 (*b*), 24 (*c*), 36 (*d*), 48 (*e*) and 60 (*f*) hours post-feeding. au arbitrary units; m/z mass-to-charge ratio

**Fig. 4:**
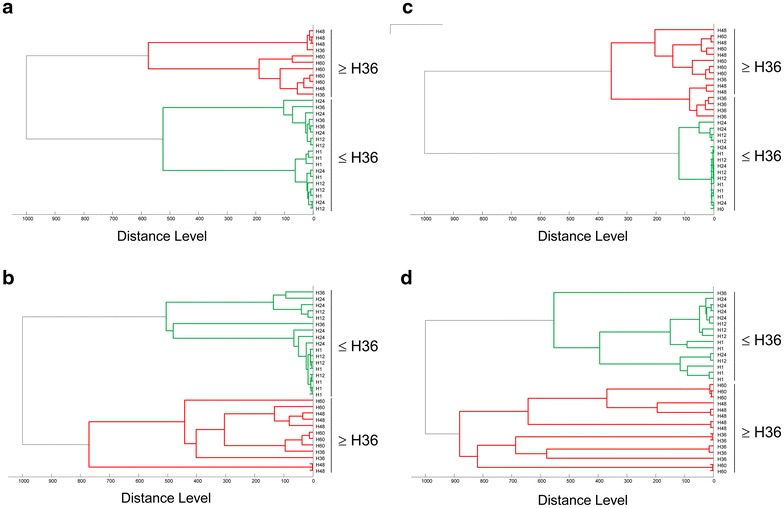
MSP dendrograms of MALDI-TOF MS spectra from *Anopheles gambiae S* abdomens collected from one to 60 h post-blood feeding. MS spectra from five distinct specimens collected at 1, 12, 24, 36, 48 and 60 h post feeding on human (**a**), horse (**b**), rabbit (**c**) and sheep (**d**) are presented. *Green* and *red* branch colour distinguishes MS spectra clustered before and after 36 h. Distance unit corresponds to the relative similarity calculated from the distance matrix

To assess the consequences of vertebrate blood protein digestion in mosquito abdomens for the MS profiles, kinetic sampling of five abdomens from *An. gambiae s.s.* engorged either on human, horse, sheep, mouse, rat, rabbit or dog blood and collected every 12 h from one to 60 h post-feeding was performed. In addition, *Ae. albopictus* specimens engorged on human blood and collected at the same points in time post-blood feeding were included, as a control group. The comparison of MS profile stability and reproducibility half-life from *An. gambiae s.s.* and *Ae. albopictus* specimens engorged on human blood according to time post-blood feeding, will be informative to assess the consequences of mosquito species on blood-source determination. Representative MS spectra from *An. gambiae s.s.* specimens engorged on human blood and kinetically collected post-blood feeding are shown in Fig. 3. Comparison of MS profiles with Flex analysis software indicated reproducibility of the spectra from one to 24 h post-blood feeding, with alteration of the MS profiles occurring 36 h post-blood feeding. Clustering analysis based on resulting MS profiles supported clear modification of these spectra occurring at 36 h post-blood feeding, and the MS profile change was observed at 36 h post-blood feeding regardless of the blood source or mosquito species (Fig. 4).

**Fig. 5:**
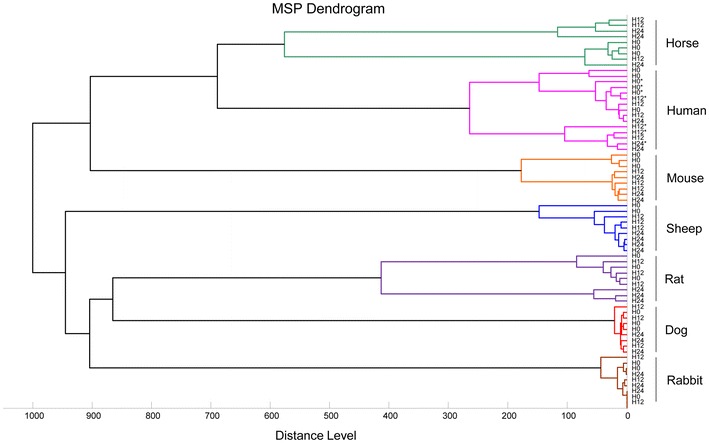
Dendrogram of MALDI-TOF MS spectra from mosquito abdomens collected one, 12 and 24 h post-blood feeding. Each time point per host blood feeding is represented by three distinct specimens. Blood meal host origin is indicated at the* right* part of the* graph* and the same color code as Fig. 3 was used. An *asterisk* (*) distinguishes *Ae. albopictus* from *An. gambiae S* specimens. Distance unit corresponds to the relative similarity calculated from the distance matrix

To visualize the specificity of MS profiles according to blood meal sources for freshly engorged specimens (i.e., <24 h), MS protein profiles from three specimens per condition described above were used to generate a dendrogram (Fig. 5). Clustering analysis revealed a gathering on distinct branches of mosquitoes according to host blood feeding origin independently of mosquito species tested. Moreover, for mosquitoes engorged on the same host, intertwining occurred between the different collection points in time, suggesting a low intra-group diversity of engorged mosquito abdomen spectra from 1 to 24 h post-blood feeding.

### MS reference spectra database creation and validation step

The MS spectra from the 69 specimens used for clustering analysis were loaded into MALDI-Biotyper v.3.0. (Bruker Daltonics, Germany) to create a database called the trophic preference (TP) database (Table 1). Then, 158 specimens including *An. gambiae s.s.* engorged on human, horse, sheep, mouse, rat, rabbit or dog blood and *Ae. albopictus* engorged on human blood and collected every 12 h from 1 to 60 h, were subject to MALDI-TOF MS analysis. The resulting spectra were queried against the TP Database, yielding 100 % correct identification of the origin of the host blood meal for specimens collected less than 24 h following their engorgement. The log score values (LSVs) for these freshly engorged specimens were greater than 2.0 (Table 1).

**Table 1:** Mosquitoes used for reference database of MALDI-TOF spectra establishment and blind tests according to post-blood feeding period and blood meal source

Mosquito species^a^	Source of blood meal	Post-blood feeding period (hours)	Number of specimens used to create the database^b^	Number of specimens used for blind test	High LSVs obtained from blind tests against database^c^	Vertebrate species identification of blood origin^d^
*An. gambiae S*	Human	1	3	2	[2.540–2.624] (2)	Human
*An. gambiae S*	Human	12	3	2	[2.799–2.854] (2)	Human
*An. gambiae S*	Human	24	3	2	[2.725–2.798] (2)	Human
*An. gambiae S*	Human	36	0	5	[2.053–2.229] (3)	Human
					[1.153–1.711] *(2)*	/
*An. gambiae S*	Human	48	0	5	[1.155–1.327] *(5)*	/
*An. gambiae S*	Human	60	0	5	[0.900–1.489] *(5)*	/
*Ae. albopictus*	Human	1	3	2	[2.743–2.799] (2)	Human
*Ae. albopictus*	Human	12	3	2	[2.759–2.863] (2)	Human
*Ae. albopictus*	Human	24	3	2	[2.329–2.924] (2)	Human
*Ae. albopictus*	Human	36	0	5	[1.803–1.868] (2)	Human
					[1.086–1.689] *(3)*	/
*Ae. albopictus*	Human	48	0	5	[1.056–1.752] *(5)*	/
*Ae. albopictus*	Human	60	0	5	[1.101–1.225] *(5)*	/
*An. gambiae S*	Horse	1	3	2	[2.712–2.885] (2)	Horse
*An. gambiae S*	Horse	12	3	2	[2.683–2.859] (2)	Horse
*An. gambiae S*	Horse	24	3	2	[2.607–2.726] (2)	Horse
*An. gambiae S*	Horse	36	0	5	[2.172–2.481] (2)	Horse
					[1.185–1.302] *(3)*	/
*An. gambiae S*	Horse	48	0	5	[1.152–1.375] *(5)*	/
*An. gambiae S*	Horse	60	0	5	[1.141–1.403] *(5)*	/
*An. gambiae S*	Sheep	1	3	2	[2.532–2.541] (2)	Sheep
*An. gambiae S*	Sheep	12	3	2	[2.497–2.563] (2)	Sheep
*An. gambiae S*	Sheep	24	3	2	[2.464–2.478] (2)	Sheep
*An. gambiae S*	Sheep	36	0	5	[1.573–1.689] *(5)*	/
*An. gambiae S*	Sheep	48	0	5	[1.174–1.304] *(5)*	/
*An. gambiae S*	Sheep	60	0	5	[1.221–1.671] *(5)*	/
*An. gambiae S*	Mouse	1	3	2	[2.371–2.593] (2)	Mouse
*An. gambiae S*	Mouse	12	3	2	[2.455–2.476] (2)	Mouse
*An. gambiae S*	Mouse	24	3	2	[2.363–2.530] (2)	Mouse
*An. gambiae S*	Mouse	36	0	5	[0.984–1.234] *(5)*	/
*An. gambiae S*	Mouse	48	0	/	[–]	
*An. gambiae S*	Mouse	60	0	/	[–]	
*An. gambiae S*	Rat	1	3	2	[2.252–2.612] (2)	Rat
*An. gambiae S*	Rat	12	3	2	[2.285–2.360] (2)	Rat
*An. gambiae S*	Rat	24	3	2	[2.298–2.555] (2)	Rat
*An. gambiae S*	Rat	36	0	5	[1.339–1.681] *(5)*	/
*An. gambiae S*	Rat	48	0	5	[1.333–1.621] *(5)*	/
*An. gambiae S*	Rat	60	0	5	[1.154–1.343] *(5)*	/
*An. gambiae S*	Rabbit	1	3	2	[2.487–2.723] (2)	Rabbit
*An. gambiae S*	Rabbit	12	3	2	[2.524–2.733] (2)	Rabbit
*An. gambiae S*	Rabbit	24	3	2	[2.486–2.497] (2)	Rabbit
*An. gambiae S*	Rabbit	36	0	5	[1.189–1.633] *(5)*	/
*An. gambiae S*	Rabbit	48	0	5	[1.166–1.487] *(5)*	/
*An. gambiae S*	Rabbit	60	0	5	[1.245–1.504] *(5)*	/
*An. gambiae S*	Dog	1	3	2	[2.621–2.802] (2)	Dog
*An. gambiae S*	Dog	12	3	2	[2.590–2.651] (2)	Dog
*An. gambiae S*	Dog	24	3	2	[2.589–2.640] (2)	Dog
*An. gambiae S*	Dog	36	0	5	[2.590] (1)	/
*An. gambiae S*	Dog				[1.515–1.627] *(4)*	/
*An. gambiae S*	Dog	48	0	5	[1.279–1.604] *(5)*	/
*An. gambiae S*	Dog	60	0	5	[1.248–1.516] *(5)*	/
*An. gambiae S*	Non-engorged	/	3			
*Ae. albopictus*	Non-engorged	/	3			
Total			78	158		

^a^Mosquitoes were collected from 1 to 60 h following blood meals and their abdomen protein extracts were submitted to MALDI-TOF MS

^b^Database was composed of MS spectra from abdomen protein extract of *An. gambiae S* fed either on human, horse, sheep, mouse, rabbit or dog bloods, and from *Ae. albopictus* specimens fed on human and collected every 12 from 1 to 24 h

^c^Into brackets are indicated the number of specimens included in each range of LSVs (upper and lower than 1.8), MS results with LSVs lower than 1.8 are italicized

^d^Vertebrate species blood origin are indicated only for specimens with LSVs upper than 1.8. LSVs, log score values

At 36 h post-blood feeding, LSVs were either greater than or less than 1.8. When LSVs were greater than 1.8, correct identification of the host blood meal origin was obtained. Conversely, when LSVs were lower than 1.8, host species identification was more hazardous with less than 50 % correct. Concerning the query of MS spectra from specimens analysed at 48 and 60 h post-engorgement, LSVs ranged from 0.900 to 1.681 and correct identification dropped to less than 30 %. Collectively, for correct identification of host blood meal origins, LSVs following the MS query against the TP Database should be greater than 1.8.

### Effect of sample storage conditions on protein spectra

**Fig. 6:**
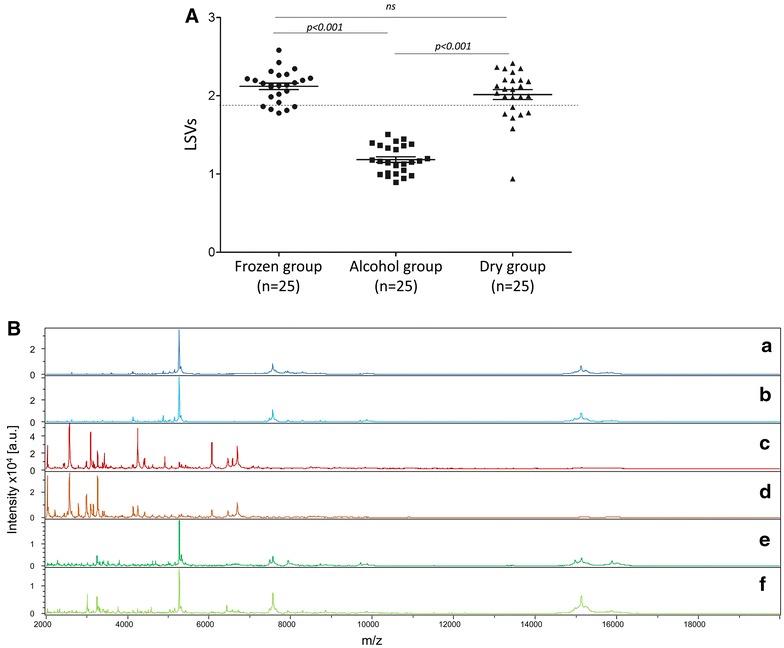
Effect of sample storage conditions on the spectra quality. **A** Comparison of LSVs obtained following reference TP database query with MS spectra of *An. gambiae S* fed on human blood, collected 12 h post-feeding and stored from 1 to 8 weeks, at −20 °C (frozen group); in 70 % ethanol at RT (alcohol group) or in a tube containing silicagel at RT (dry group). *Dashed line* represent the threshold value for relevant identification (LSVs >1.8). LSV, log score value. **B** Alteration of MALDI-TOF MS profiles from *An. gambiae S* specimens fed on human blood according to preservation mode. MS profiles from engorged *An. gambiae S* abdomens stored at −20 °C during 1 (*a*) or 4 (*b*) weeks; in 70 % ethanol at RT during 1 (*c*) or 4 (*d*) weeks; or in a tube containing silica gel at RT during 1 (*e*) or 4 (*f*) weeks. au arbitrary units; m/z mass-to-charge ratio

Abdominal protein extracts of five engorged mosquitoes collected 12 h post-human blood feeding from the frozen group, the alcohol group and the dry group, stored for 1–8 weeks were submitted for MALDI-TOF MS analysis to evaluate their suitability for determination of blood host origins. For the frozen group, the query of the TP database with the spectra of the 25 engorged specimens were all correctly identified (100 %) for the host blood origin. Their spectra possessed a high intensity and reproducibility throughout the duration of storage. Moreover, LSVs ranged from 1.778 to 2.583, among which 96 % (n = 24) possessed an LSV greater than 1.8 (Fig. 6A). Similarly, for the dry group, with the exception of one sample with a low MS spectra quality giving an LSV of 0.94, correct host blood origins were obtained with LSVs ranging from 1.58 to 2.42 for all other samples. The LSV threshold was reached for 19 samples (i.e., LSVs >1.8). For the alcohol group, none of the spectra tested reached the threshold value, with LSVs ranging from 0.89 to 1.51. Correct identification of host blood origins was obtained for 14 samples (56 %). Comparison of the spectra according to mode of storage revealed that the alcohol storage mode drastically modified MS profiles (Fig. 6B).

Independently of the sampling time (i.e., seven, 14, 21, 28, and 56 days of storing), the LSVs differed significantly according to storage mode (Kruskal–Wallis test, *p* < 0.001, Fig. 6A). Pair-wise comparisons indicated the alcohol storage mode led to significant decrease in LSVs compared to the frozen (*p* < 0.001, Mann–Whitney test) or dry (*p* < 0.001, Mann–Whitney test) modes. No significant difference was detected between the frozen and dry storage modes (*p* > 0.05, Mann–Whitney test).

## Discussion

To determine vectorial capacity as an epidemiological parameter of vector-borne diseases, mosquito identification and blood meal source determination are essential. The recent application of the MALDI-TOF MS method for arthropod identification including mosquitoes at adulthood [8, 9, 11, 27] and immature developmental stages [12, 28] offered new opportunities for monitoring mosquito populations and the detection of invasive species [11]. However, at the adult stage, mosquito abdomens containing gut protein mixtures and varying according to vertebrate blood source and post-ingestion delay, were generally excluded from MS analysis to prevent protein spectra heterogeneity from hampering arthropod species identification [9, 27]. Thus, it was hypothesized that the deleterious effect of abdominal protein varies in MS mosquito species identification, and could be advantageous for determining mosquito blood meal sources.

The present study demonstrated that reproducible MS spectra were obtained from abdomen protein extracts of *Anopheles* that were freshly engorged (i.e., ≤24 h) on the same vertebrate host. Moreover, the generation of distinct MS profiles from abdomens of *Anopheles* freshly engorged on different types of vertebrate blood indicated inter-vertebrate blood spectra specificities, also for closely-related mammalian species like mouse and rat. These data suggests the potential use of this new method for determining the origins of blood feeding. The overlap of several MS peaks obtained from human blood and *An. gambiae s.s.* specimens engorged on human blood suggested that a large part of the MS protein profiles of *An. gambiae s.s.* specimens engorged on human blood correspond to human blood proteins. The MS profiles obtained from *Ae. albopictus* specimens also engorged on human blood supported this last claim and sustained specificity of the blood MS profiles independently of the mosquito species. Clustering analysis confirmed that the primary determinant for MS profiles was the origin of the blood meal, independent of the post-ingestion delay (if less than 24 h) and of mosquito species. Further experiments are needed to confirm the reproducibility of MS spectra from mosquitoes of different species and genera engorged on the same vertebrate hosts, which will simplified TP database increments for further identification of mosquitoes from others species engorged on same host.

The determination of vertebrate blood protein half-life in the mosquito gut was a critical parameter for successfully identifying blood meal sources by MALDI-TOF MS. Effectively, immediately after mosquito blood feeding, blood meal digestion occurred, leading to both host DNA and protein degradation [20]. Kinetic analysis revealed that blood MS profiles of engorged mosquitoes were stable until 24 h post-blood ingestion and the chance to identify blood sources fell dramatically 36 h post-ingestion [20, 21]. Blood source determination by the ELISA method, also targeting vertebrate blood proteins, was generally performed on freshly engorged specimens due to the quick digestion of protein contained in the gut [18, 29, 30] whereas, with molecular biology, identification of the blood meal origin is still possible up to 36 h post-feeding, corroborating previous studies [20, 21]. Although the window of time for blood meal source determination by MALDI-TOF MS was shortened compared to molecular biology, the rapidity and low cost of the reagents made this proteomic method a financial and reliable competitive strategy. Effectively, a recent study reported tick identification at the species level in less than 1 h using MALDI-TOF MS tools [8]. However, the relative elevate price of the machine, approximately 130,000 USD for catalogue price of the Microflex LT MALDI-TOF Mass Spectrometer and associated softwares (Bruker Daltonics), could be an obstacle of the implement of this innovative tool in a laboratory.

To increase the time frame for MS blood meal source analysis, detection of residual blood meal proteins could be a complementary approach. In this case, sample treatment with a tryptic enzyme was needed prior to exploring peptide mass fingerprinting by MS. Two studies using this method succeeded in detecting blood meal peptide residues even months after blood meal feeding in *Ixodidae* [31, 32]. These works highlighted that host protein residues can persist beyond DNA degradation. Although they are very sensitive, a major limitation of these proteomic approaches is that they require many steps for sample preparation and the use of tandem mass spectrometry apparatus. Therefore, this strategy currently remains poorly suited for fast detection of host vector preferences.

Blind tests against the TP database using MS spectra from engorged mosquito abdomens show correct identification of blood meal sources for 100 % of the freshly engorged specimens (i.e., ≤24 h) with LSVs greater than two. At later points in time, the rate of correct identification decreased, reflecting the degradation of vertebrate blood proteins. However, at 36 h post-blood ingestion, correct blood meal source identification was obtained for all spectra reaching 1.8 of LSV. Based on these results, a threshold of 1.8 from LSVs against the TP database query should be obtained to be considered a reliable identification for determination of host blood meal origins. For mosquito identification using legs with MALDI-TOF MS, a threshold of 1.8 was also previously defined [9]. Interestingly, MALDI-TOF MS analysis requires only a small fraction of the sample, after which it is then possible to validate identification by molecular methods. Moreover, crushing of the mosquito abdomens was performed in water, which is compatible with further cross-validation of the host blood source by molecular biology [33].

MALDI-TOF MS will subsequently be applied to decipher *Anopheles*-human contact in malaria-endemic areas by priority on the African continent, where malaria remains a primary cause of mortality, with approximately 600,000 deaths per year among which 91 % occurred in Africa [34]. The recent acquisition of a MALDI-TOF MS apparatus in Dakar, Senegal [35] made possible the transfer of expertise and the TP database for field applications to determine anopheline fauna trophic behaviour. To achieve this transfer, and because laboratories are generally far from *Anopheles* field collection sites, different preservation were tested. The frozen mode was the best method for sample storage with a sensitivity of 96 %, taking into account only spectra queries reaching an LSV threshold of 1.8 and a specificity of 100 %. For the dry mode, a sensitivity and specificity of 79 and 100 % were respectively recorded, suggesting that this storage method could be applied in areas where frozen sample preservation is not possible. Moreover, the absence of significant LSV variations according to duration of storage up to at least 2 months suggested that the frozen and dry preservation modes prevented significant vertebrate blood protein degradation. Conversely, a significant LSV decrease was observed for samples stored in 70 % ethanol with none reaching the 1.8 threshold value (sensitivity 0 %). The changes in peak position and disappearance of higher weight masses are phenomena repeatedly reported for arthropods stored in ethanol and further analysis by MALDI-TOF MS [36, 37]. This storage mode did not appear well adapted to MS identification of mosquito blood sources.

More recently, the proof-of-concept for dual identification of tick species and associated pathogens by applying the MALDI-TOF MS approach was demonstrated using either tick legs [38, 39] or tick haemolymph [40]. The possibility of identifying micro-organisms inside the vectors offers new perspectives for *Plasmodium sp.* monitoring in *Anopheles* by this proteomic approach. Then, it is likely that in the next future, this unique tool could be useful for mosquito species identification, blood source determination and detection of malaria infections. The characterization of these three parameters will enable better understanding human/*Anopheles*/*Plasmodium* interactions.

## Conclusions

The present study demonstrated that MALDI-TOF MS profiling of blood meals in mosquito abdominal protein extracts can be used for rapid identification of trophic preference independently of the mosquito species. The reproducibility and specificity of MS spectra are important parameters to determine feeding patterns; however, the protein profile stability up to 24 h post-feeding limits the application of this proteomic approach for freshly engorged mosquitoes. The storage mode of engorged mosquitoes is also a critical factor for the success of further MALDI-TOF MS blood meal source identification. Finally, the determination of blood meal sources from mosquito abdomens using MALDI-TOF MS provides rapid and responsive solutions in the surveillance, risk assessment and prevention of vector-borne diseases, by identification of not only arthropod species, but also trophic preference for specimens collected in the field during entomological surveys. Nevertheless, the addition of reference MS spectra from other mammalian hosts, like domestic animals, livestock and monkeys, is necessary for confirming the specificity of the MS profiles according to host blood-feeding and is indispensable for assessing this tool with engorged anophelines specimens collected in field conditions.
